# Draft Genome Sequences of Two Cultivable Strains of the Bacterial Symbiont Serratia symbiotica

**DOI:** 10.1128/MRA.01579-19

**Published:** 2020-03-05

**Authors:** François Renoz, Jérôme Ambroise, Bertrand Bearzatto, Patrice Baa-Puyoulet, Federica Calevro, Jean-Luc Gala, Thierry Hance

**Affiliations:** a Earth and Life Institute (ELI), Biodiversity Research Centre, Université Catholique de Louvain, Louvain-la-Neuve, Belgium; b Center for Applied Molecular Technologies (CTMA), Institut de Recherche Expérimentale et Clinique (IREC), Université Catholique de Louvain, Woluwe-Saint-Lambert, Belgium; c Univ Lyon, INSA-Lyon, INRAE, BF2i, UMR0203, Villeurbanne, France; University of Maryland School of Medicine

## Abstract

Serratia symbiotica, one of the most frequent symbiont species in aphids, includes strains that exhibit various lifestyles ranging from free-living to obligate intracellular mutualism. Here, we report the draft genome sequences of two strains, namely, 24.1 and Apa8A1, isolated from aphids of the genus *Aphis*, consisting of genome sizes of 3,089,091 bp and 3,232,107 bp, respectively. These genome sequences may provide new insights into how mutualistic interactions between bacteria and insects evolve and are shaped.

## ANNOUNCEMENT

Serratia symbiotica is one of the most common inherited endosymbionts found in aphids. It constitutes a suitable symbiotic bacterium model for understanding the evolution of bacterial mutualism in insects, as it includes a wide diversity of strains displaying various associated phenotypes and lifestyles, ranging from free-living to obligate intracellular mutualism (1–5). Some of these strains have been isolated and successfully cultured on artificial rich medium (6, 7), which has made it possible to conduct experiments to study their associated effects on newly infected host aphids (8–10).

We report here the draft genome sequences of two *S. symbiotica* strains displaying free-living capacities, namely, strain 24.1, previously isolated from the black bean aphid Aphis fabae, and strain Apa8A1, previously isolated from the sage aphid Aphis passeriniana (7). Bacterial cultures were started from single colonies and grown in 863 medium at 20°C (6). Genomic DNA was extracted using the DNeasy blood and tissue kit (Qiagen). Whole-genome libraries were prepared from 1 ng DNA using the Nextera XT DNA library preparation kit (Illumina, San Diego, CA, USA), according to the manufacturer’s instructions. As previously described (11), libraries were then sequenced on the MiSeq platform (Illumina) using paired-end sequencing. More than 115,000 paired-end reads of 2 × 300 bp were obtained for both strains. Paired-end reads were quality checked by FastQC (http://www.bioinformatics.babraham.ac.uk/projects/fastqc/) and assembled *de novo* using the SPAdes v.3.11.1 algorithm (12) with default settings to generate a draft genome sequence. Quality assessment for genome assemblies was carried out using QUAST 4.5 (13). The annotation of both genomes was performed using the Prokaryotic Genome Annotation Pipeline (PGAP) with default settings in order to highlight the main features (14). The draft genome of *S. symbiotica* strain 24.1 consists of 146 contigs with a total length of 3,089,091 bp, an *N*_50_ value of 69,946 bp, an average depth of 116×, and a G+C content of 51.4% and contains 2,619 coding DNA sequences (CDSs) (with proteins), 335 pseudogenes, 7 complete rRNA genes, 62 tRNA genes, and 1 CRISPR array. The draft genome of *S. symbiotica* strain Apa8A1 consists of 184 contigs with a total length of 3,232,107 bp, an *N*_50_ value of 54,840 bp, an average depth of 110×, and a G+C content of 51.9% and contains 2,811 CDSs (with proteins), 343 pseudogenes, 7 complete rRNA genes, 63 tRNA genes, and 1 CRISPR array.

Genome analyses that were performed on other strains of *S. symbiotica* have shown that this symbiont holds genomes of contrasting sizes and features related to different lifestyles (1, 5). *S. symbiotica* strains associated with aphid species of the subfamily Lachninae are nutritional co-obligate partners with the obligate symbiont Buchnera aphidicola and exhibit highly eroded genomes (1, 15–19). In the pea aphid Acyrthosiphon pisum (subfamily Aphidinae), *S. symbiotica* includes intracellular strains of a facultative nature showing moderately reduced genome sizes (3, 20–22). In addition to co-obligate and facultative strains, *S. symbiotica* also includes strains capable of growing independently from their host aphid on an artificial rich medium (6, 7). The genomic features of the strain CWBI-2.3^T^, isolated from *A. fabae*, suggest that it may represent a missing link in the evolution of a free-living lifestyle toward a host-dependent lifestyle (1, 2, 6, 7). The genome sizes of strains 24.1, Apa8A1, and CWBI-2.3^T^ are similar (∼3 to 3.5 Mb) (2). More in-depth genomic analyses are under way to decipher the metabolic capabilities of these cultivable strains and their genetic determinants potentially involved in host colonization.

### Data availability.

This whole-genome shotgun project has been deposited at DDBJ/ENA/GenBank under the accession numbers WSPN00000000 and WSPO00000000. Raw sequence reads have been deposited in the NCBI Sequence Read Archive under BioProject numbers PRJNA595064 and PRJNA595070 and run numbers SRR10882921 and SRR10882920.
